# Effects of 4 major brain protection strategies during aortic arch surgery: A protocol for a systematic review and network meta-analysis using Stata

**DOI:** 10.1097/MD.0000000000011448

**Published:** 2018-07-06

**Authors:** Shulei Fan, Daoxing Wang, Chun Wu, Zhengxia Pan, Yonggang Li, Yong An, Hongbo Li, Gang Wang, Jiangtao Dai, Quan Wang

**Affiliations:** a Chongqing Medical University; b Department of Respiratory Medicine, Second Affiliated Hospital of Chongqing Medical University; c Department of Cardiothoracic Surgery, Children's Hospital of Chongqing Medical University, Ministry of Education Key Laboratory of Child Development and Disorders; d China International Science and Technology Cooperation Base of Child Development and Critical Disorders; e Chongqing Key Laboratory of Pediatrics, Chongqing Medical University, Chongqing, China.

**Keywords:** aortic arch, brain protection, network meta-analysis, perioperative mortality, permanent neurological deficits

## Abstract

**Background:**

Reliable brain protection during aortic arch surgery remains a formidable surgical challenge. Various cerebral protection techniques have been used in the clinic; however, there is no consensus regarding which strategy is best. We will perform a network meta-analysis (NMA) focusing on the permanent neurological deficits (PND) and perioperative mortality associated with 4 major brain protection strategies used during aortic arch surgery.

**Methods:**

We will perform a literature search of MEDLINE, EMBASE, Cochrane Library, and PubMed databases. The primary outcomes of interest in this analysis will be PND and perioperative mortality. Inconsistencies in the NMA will be evaluated with global and local approaches. Network rank and surface under the cumulative ranking curve (SUCRA) analyses will be performed to evaluate and identify the superiority of different brain protection techniques.

**Results:**

This study is ongoing and will be submitted to a peer-reviewed journal for consideration of publication.

**Conclusions:**

Our study will increase understanding of 4 major brain protection strategies during aortic arch surgery and be helpful to clinicians using NMA in their studies.

## 1 Introduction

Aortic arch surgery was widely considered a forbidden field for surgeons before the 1950s.^[1]^ With the use of a mechanical heart and lungs and hypothermic techniques^[2]^ for cardiac surgery, Debakey was the first surgeon to report his experience with total excision of the aortic arch due to an aneurysm thus breaking this restriction in the field of cardiac surgery. Various techniques, such as antegrade cerebral perfusion (ACP)^[3]^ and retrograde cerebral perfusion (RCP),^[4]^ have been subsequently developed to provide better cerebral protection during aortic arch surgery. Deep hypothermic circulatory arrest (DHCA) alone, DHCA with ACP (DHCA + ACP), DHCA with RCP (DHCA + RCP), and moderate hypothermic circulatory arrest (MHCA) with ACP (MHCA + ACP) are currently the 4 major brain protection strategies applied in the clinic.^[5,6]^

However, even with the development of cerebral perfusion techniques, cannulation sites and hypothermic techniques, permanent neurological deficits (PND) and perioperative mortality are still not rare.^[7]^ Cerebral perfusion techniques can provide extra oxygenated blood flow to the brain,^[8]^ but may be associated with additional vascular injury.^[9]^ RCP is a simple cerebral perfusion technique that is associated with lower risks^[10]^ than ACP; however, animal studies have indicated that blood flow to the brain is negligible.^[11,12]^ The most suitable temperature for hypothermic circulatory arrest with cerebral perfusion is unclear. Deep hypothermia more efficiently reduces the cerebral metabolic rate^[13]^ but causes more inflammation, acidosis, vasospasm, and ischemia-reperfusion injury than moderate hypothermia.^[14]^

Network meta-analysis (NMA) is a new method that employs Bayesian statistical theory. In this NMA, we will be able to perform multiple comparisons and rank their effects.^[15]^ Hence, we will perform a systematic review and NMA to increase our understanding of brain protection strategies during aortic arch surgery. To make the protocol more scientific, we will evaluate the reliability of the NMA with critical assessments of the quality of the included studies, inconsistency and publication bias.^[16]^

All analyses will be performed with Stata (version 14.0, Stata Corp, College Station, TX), and we will provide all the codes. Hence, the protocol will be helpful to clinicians using NMA in their studies.

## 2 Methods

### 2.1 Design and registration

This systematic review and meta-analysis will be performed in accordance with the Preferred Reporting Items for Systematic Reviews and Meta-Analyses (PRISMA) statement^[17]^ and the PRISMA Extension Statement for Reporting of Systematic Reviews Incorporating Network Meta-analyses of Health Care Interventions.^[18]^ The study protocol has been registered at the International Prospective Register of Systematic Reviews (PROSPERO, CRD42018094824).

### 2.2 Ethics and dissemination

Ethics approval and patient consent are not required because this study is a NMA based on the published literature. The results of this study will be submitted to a peer-reviewed journal.

### 2.3 Eligibility criteria

The 4 brain protection strategies that will be included in the NMA are DHCA, DHCA + ACP, DHCA + RCP, and MHCA + ACP. DHCA is defined as initiation of circulatory arrest coinciding with a nasopharyngeal temperature of 14.1 to 20°C. MHCA is defined as a circulatory arrest with a temperature of 20.1 to 28°C.^[5]^ For repeated research objectives, only the study with the most detailed information will be included. The affiliations of the authors will be carefully checked to avoid possible duplicates, especially for multicenter studies.

### 2.4 Search strategy

We will search MEDLINE via Ovid (from 1946 through December 2017), EMBASE via Ovid (from 1980 through December 2017), the Cochrane Library database via Ovid (Cochrane Central Register of Controlled Trials; through December 2017), and PubMed (through December 2017). The complete text that will be used to search PubMed is as follows: ((((((((circulatory arrest) OR cerebral perfusion) OR antegrade) OR hypothermic) OR retrograde)) AND ((((((((((thoracic aorta) OR aortic arch) OR arch, aortic) OR arch of the aorta) OR aorta, descending) OR descending aorta) OR aortas) OR arch) OR aorta)))) AND (“1900”[Date—Publication]: “2017”[Date—Publication]). We will consider all potentially eligible studies without language restrictions. We will also perform hand searches of the bibliographies and internet searches of unpublished studies in the form of posters or abstracts.

### 2.5 Study selection

Three pairs of investigators will independently review the eligible scientific reports, extract the data and assess the quality of the studies. Any discrepancies will be resolved by consensus and arbitration by a panel of investigators within the review team.

Studies will be included in the analyses if they meet the following criteria: they were randomized controlled trials (RCTs) or observational cohort studies (OCS); they involved patients undergoing aortic arch surgery; they used at least two of the 4 brain protection strategies mentioned above; and at least 1 major outcome was clearly mentioned.

Duplicate reports, case reports, reviews, letters with no exact data, meta-analyses, and animal studies will be excluded. A flow diagram of the searching and screening process will be made.

### 2.6 Data collection process and quality assessment

The quality of OCS will be independently evaluated by each investigator using the Newcastle-Ottawa quality assessment scale (NOS), and a final score greater than 6 will be regarded as indicative of high quality.^[19]^ The 2009 Updated Method Guidelines for Systematic Reviews in the Cochrane Back Review Group will be used for quality assessment of RCTs, and studies will be rated as having a “low risk of bias” when at least 6 of the 12 criteria are met.^[20]^ EndNote and manual entry will be used to merge the retrieved citations and eliminate duplications. The following variables will be extracted: author name(s), publication year, publication journal, study type, country where the study was conducted, methods of the brain protection strategies, total sample size, operative time, disease type, surgery type, mortality rates, incidence of PND and surgical approaches.

### 2.7 Outcomes

PND and mortality will be the primary outcomes. PND are defined as stroke or persistent focal neurologic dysfunction, often accompanied by changes in brain imaging.^[21]^ Mortality is defined as death that occurred intraoperatively, within the same admission postoperatively, or by 30 days postoperatively.^[7]^ Temporal were neurological deficits (TND) and acute kidney injury (AKI) will also be recorded as secondary outcomes.

### 2.8 Data synthesis and analysis

#### 2.8.1 Data synthesis

Data for PND and mortality will be obtained from individual studies, and risk ratios (RRs), weights, 95% confidential intervals (CIs), and 95% predicted intervals (PrI) will be calculated.

#### 2.8.2 Geometry of the network

Network geometry will be performed to show the interactions among the studies included in the NMA. The contributions of direct comparisons in the network will be demonstrated as a contribution plot for the network.

#### 2.8.3 Inconsistencies in the NMA

Inconsistencies in the NMA will be evaluated with global and local approaches. The global approach via the Wald test will be used as a measurement of overall inconsistency; the level of inconsistency will be computed according to the type of between-treatment comparisons for all cases. Loop and pairwise comparisons will be used as local approaches to assess for inconsistency. In the local approach, each treatment will be individually examined, and the outcomes of direct and indirect comparisons will be statistically tested. For more precise statistical results, a random effects model will be used, and consistency will be defined as a *P*-value >.1 instead of .05.

#### 2.8.4 Network meta-analysis

A consistency model will be used in the NMA only when inconsistency is not found by both global and local tests. The treatment effect of the NMA will be shown in a forest plot for pairwise comparisons of the network. Network rank and surface under the cumulative ranking curve (SUCRA) analyses will be performed to evaluate and determine the superiority of different brain protection techniques.

#### 2.8.5 Publication bias

A network funnel plot will be created to check for publication bias in the NMA.

### 2.9 Quality of evidence for primary outcomes

The quality of evidence for the primary outcomes will be assessed as 4 levels (high level, moderate level, low level, and very low level) by the Grading of Recommendations Assessment, Development and Evaluation (GRADE) for NMA.^[22]^ Risk of bias, inaccuracy, inconsistency, indirectness, and publication bias will decrease the level of evidence. Large magnitude of effect, opposing plausible residual bias or confounding and dose–response gradient are 3 factors can increase quality. The results will be shown as Summary of Findings (SoF) tables.

### 2.10 Code of NMA by Stata

#### 2.10.1 Installation

ssc install metan

ssc install network

ssc install mvmeta

ssc install metareg

ssc install metafunnel

#### 2.10.2 Data synthesis and setting

network setup r n, study(id) trt(t) rr ref(1)

network convert pairs

gen invvarES=1/(_stderr^2)

#### 2.10.3 Geometry of the network

networkplot _t1 _t2, edgew(invvarES) edgesc(1.2) asp(0.8) lab(DHCA “DHCA+ACP” DHCA+RCP “MHCA+ACP”)

#### 2.10.4 Contributions of direct comparisons

netweight _y _stderr _t1 _t2,asp(0.7)

#### 2.10.5 Inconsistency of NMA

Global approach by overall inconsistency:

network convert augment

network meta inconsistency

Local approach by loop comparison:

network convert pairs

ifplot _y _stderr _t1 _t2 id,eform

Local approach by pairwise comparison:

network convert augment

network sidesplit all,tau

#### 2.10.6 Network meta-analysis

Model establishment:

network meta c (consistency model)

network meta I (inconsistency model)

Pairwise comparisons of network:

intervalplot, pred null(1) lab(DHCA “DHCA+ACP” DHCA+RCP “MHCA+ACP”) sep marg(0 20 5 5) xlab(0.5 1 2 3 4 5) eform

Network rank:

network rank min, seed(50000) all bar cumul reps(10000)

SCURA rank:

network rank min, all zero gen(prob)

network rank min, all zero predict gen(predprob)

sucra prob∗, compare(predprob∗) name(“Estimated Probabilities” “Predictive Probabiliites”) lab(DHCA “DHCA+ACP” DHCA+RCP “MHCA+ACP”)

#### 2.10.7 Publication bias

network convert pairs

netfunnel _y _stderr _t2 _t1, bycomparison addplot(lfit _stderr _ES_CEN)

## 3 Discussion

To our knowledge, this would be the first NMA in the area and we will perform a systematic review of brain protection strategies in aortic arch surgeries. We hope our work can be a stage to close to the answer, although it may not conquer the problem right now.

## Author contributions

**Data curation**: Shulei Fan, Daoxin Wang, Gang Wang, Jiangtao Dai, Hongbo Li, and Zhengxia Pan.

**Formal analysis**: Daoxin Wang, Zhengxia Pan, and Yonggang Li.

**Funding acquisition**: Yong An.

**Investigation**: Quan Wang and Chun Wu.

**Methodology**: Quan Wang, Daoxin Wang, and Yong An.

**Software**: Shulei Fan and Chun Wu.

**Writing – original draft**: Quan Wang and Shulei Fan.

**Writing – review & editing**: Quan Wang and Shulei Fan.
